# Effectiveness and safety of eltrombopag in connective tissue disease patients with refractory immune thrombocytopenia: a retrospective study

**DOI:** 10.1093/rap/rkae029

**Published:** 2024-03-04

**Authors:** Xiangpin Jiang, Xiaoming Shu, Yongpeng Ge

**Affiliations:** Department of Rheumatology, Jining No. 1 People’s Hospital, Jining, China; Department of Rheumatology, Key Laboratory of Myositis, China-Japan Friendship Hospital, Beijing, China; Department of Rheumatology, Key Laboratory of Myositis, China-Japan Friendship Hospital, Beijing, China

**Keywords:** connective tissue disease, immune thrombocytopenia, eltrombopag, refractory

## Abstract

**Objectives:**

We aimed to investigate the safety and effectiveness of eltrombopag for adult patients with refractory immune thrombocytopenia (ITP) secondary to connective tissue disease (CTD).

**Methods:**

This is a single-centre, retrospective cohort and propensity score-matched study. Data from CTD-ITP patients treated with eltrombopag between January 2019 and January 2023 were retrospectively analysed. Baseline characteristics and follow-up information were recorded. CTD patients without ITP were matched to identify the risk factors associated with CTD-ITP performed by Logistic regression analysis.

**Results:**

Twenty patients were enrolled, including 5 systemic lupus erythematosus (SLE), 9 Sjögren’s syndrome (SS) and 6 undifferentiated connective tissue disease (UCTD). Nineteen (95%) patients were female, and the median age was 59 years. Logistic regression analysis showed that anaemia (OR = 8.832, *P *=* *0.007) was associated with increased risk of ITP, while non-erosive arthritis (OR = 0.045, *P *=* *0.001) and interstitial lung disease (OR = 0.075, *P *=* *0.031) were associated with reduced risk. Fourteen patients (70%) achieved a complete response (CR) and one (5%) achieved a partial response (PR). The median response time was 14 days. The median platelet count was 8.5 × 10^9^/l at baseline of eltrombopag and increased to 122 × 10^9^/l after 4 weeks. No adverse events were observed.

**Conclusions:**

Eltrombopag appears to be effective, safe and well-tolerated in refractory ITP patients with CTD; larger studies are needed to confirm the generalizability of these findings.

## Graphical Abstract

**Figure:**
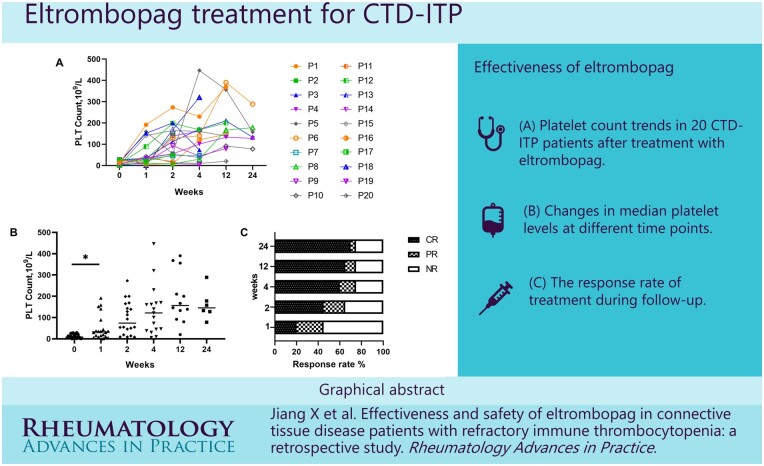

Key messages

- As a non-immunomodulatory agent, eltrombopag is an attractive alternative in the treatment of CTD-ITP.

## Introduction

Connective tissue diseases (CTD) are autoimmune disorders such as systemic lupus erythematosus (SLE), Sjögren’s syndrome (SS), anti-phospholipid syndrome (APS) and undifferentiated connective tissue disease (UCTD) [1]. These diseases share pathophysiological mechanisms that are primarily characterized by autoimmunity and immune-mediated organ dysfunction and include hematological abnormalities. Immune thrombocytopenia (ITP) is a common hematological manifestation of CTD and is classified as a secondary immune thrombocytopenia (sITP) [2]. Compared with primary ITP, the pathogenesis of ITP secondary to CTD is more complicated [3]. CTD-related thrombocytopenia can range from a slight platelet count decrease to life-threatening hemorrhage; severe thrombocytopenia independently raises the risk of mortality [4].

To increase platelet counts and avoid life-threatening hemorrhage, CTD-ITP patients are recommended to be treated with high-dose glucocorticoids and/or intravenous immunoglobulin (IVIG) as first-line therapy. Second-line therapies include rituximab, splenectomy and immunosuppressants such as ciclosporin (CsA), azathioprine (AZA), mycophenolate mofetil (MMF) and cyclophosphamide (CTX) that primarily aim to control the destruction of antibody-coated platelets [5, 6]. However, some patients fail to respond to these multimodal therapies [7]. Refractory ITP is diagnosed in cases that fail to respond to two or more treatments [5]. Approximately 10% of ITP patients show poor or absent responses to treatment and develop refractory ITP [8].

Eltrombopag is a non-peptide thrombopoietin receptor agonist (TPO-RA) that is approved for the treatment of ITP patients with an inadequate response to other therapies. It is effective in up to 80% of primary ITP cases, but the evidence remains scarce for sITP [9]. As a non-immunotherapeutic ITP therapy, eltrombopag presents an attractive approach to CTD-ITP because of the lower risk of immunosuppression [6]. However, to date, no large clinical trials have evaluated the effectiveness or safety of eltrombopag on CTD-ITP, and only studies involving case reports and small sample studies have been published [10–13]. In this study, we retrospectively reviewed data from refractory CTD-ITP patients treated with eltrombopag in our center to establish therapeutic effectiveness and safety profiles.

## Materials and methods

### Patients

This retrospective study was conducted with patients from the China-Japan Friendship Hospital from January 2019 to January 2023. In the retrospective cohort study, patients diagnosed with refractory thrombocytopenia in CTD treated with eltrombopag were eligible. The inclusion criteria were (1) diagnosis with SLE according to the European League Against Rheumatism (EULAR)/American College of Rheumatology (ACR) classification criteria for SLE (2019), SS according to ACR/EULAR Classification Criteria (2016) and UCTD according to the preliminary classification criteria by Mosca et al. [14–16]; and (2) platelet counts < 30 × 10^9^/l after treatment with two or more therapies. Patients diagnosed with APS or with anticardiolipin (ACL) antibody positive were excluded. Other exclusion criteria: severe infection, drug-associated thrombocytopenia, liver cirrhosis or hematological malignancy. To identify the risk factors associated with CTD-ITP, we also enrolled a control group of CTD patients admitted to the same hospital at the same time. The control group and the case group were matched 2:1 according to the same sex, the same primary disease. To overcome possible selection bias, propensity score-matched study was performed using the data of the case group and control group. The study protocol was approved by the institutional ethics committee of the China-Japan Friendship Hospital (IRB number 2022-KY-156). The study was performed according to the ethical principles of the Declaration of Helsinki. Informed consent was obtained from participants at the time of admission.

### Data analyses and effectiveness evaluation

Clinical data including gender, age, course of the disease, symptoms and laboratory examination results were collected from medical records. Baseline thrombocytopenia data, previous medications used for managing thrombocytopenia, bone marrow examination results and complications of CTD were obtained. For eltrombopag treatment, we noted the dose, duration of treatment, concurrent therapies, platelet counts at initiation and follow-up, and side effects. Comorbidities, clinical symptoms and laboratory tests were compared in the two CTD groups using a propensity score-matched analysis. The clinical effectiveness of drug therapy was evaluated based on platelet counts at baseline, and after 1, 2, 4, 12, and 24 weeks of eltrombopag treatment. Outcomes were categorized according to the guidelines of the International Working Group and the American Society of Hematology: complete response (CR) was platelets of ≥ 100 × 10^9^/l without bleeding, partial response (PR) was platelets of ≥ 30 × 10^9^/l that were ≥ 2× the baseline value without bleeding, non-response (NR) was platelets of ≤ 30 × 10^9^/l or < 2× the baseline value or the presence of bleeding [2]. The proportions of PR, CR and NR at each time point were also calculated.

### Statistical analysis

Group descriptive statistics were calculated for demographic and baseline characteristics. Continuous variables are reported as (IQR, interquartile range) or mean ± standard deviation (SD) as appropriate while categorical variables are expressed as count (percentage). Correlation factors of CTD complicated with ITP were analysed by single factor analysis. Logistic regression analysis was performed for statistically significant factors. Between-group comparisons were performed using Fisher’s exact test or the chi-square test for categorical variables, and the Mann–Whitney test for quantitative variables. Due to the small sample size and uneven distribution, non-parametric methods were used for statistical evaluation of data. *P *<* *0.05 was considered to indicate statistical significance. Statistical analyses were conducted with GraphPad Prism 9.0 software or SPSS 27.0 (IBM, Armonk, NY) software.

## Results

### General cohort characteristics

Data from 20 patients of East Asian ancestry with refractory CTD-ITP were included. The median age was 59 (IQR 35–69.75) years, and 19 (95%) patients were female. These patients included 5 SLE, 9 SS and 6 UCTD. Among all cases, 3 (15%) had malar rash or vasculitic skin rash, 5 (25%) had xerostomia, 8 (40%) had xerophthalmia, 4 (20%) had Raynaud’s phenomenon, and 2 (20%) had non-erosive arthritis. 15 (75%) cases had varying degrees of bleeding symptoms; one (Patient 8) had gastrointestinal bleeding, one (Patient 19) had hematuria, and 13 had skin mucosal bleeding. One patient had splenomegaly. Also, 2 (10%) had proteinuria, 1 (5%) had interstitial lung disease (ILD), and 1 (5%) had pulmonary arterial hypertension (PAH). Laboratory tests showed leukopenia in 2 (10%) patients, anaemia in 15 (75%), low complement in 9 (45%), and elevated immunoglobulin in 5 (25%)0.19 patients (95%) were positive for antinuclear antibodies (ANAs), 11 (55%) for anti-SSA antibodies, 13 (65%) for anti-Ro52 antibodies, and 5 (25%) for Anti-dsDNA antibodies. Only one patient had a completely negative antinuclear antibody spectrum, but she had dry eye and dry mouth symptoms, Schirmer’s test ≤5 mm/5 min in both eyes, and a labial gland biopsy showed focal lymphocyte infiltration, which met the classification criteria for SS. The median disease duration before eltrombopag treatment was 2 (IQR 0.73–5.75) years. In 13 patients, severe thrombocytopenia was the initial presenting manifestation of CTD. In the other 7 patients, the median disease duration before the onset of thrombocytopenia was 8 (IQR, 0.5–24) months. The median lowest platelet count was 4 (IQR, 2–5) × 10^9^/l. Before the start of eltrombopag therapy, all patients received high-dose glucocorticoids (at least 1 mg/kg/day). Of these, 10 patients had received glucocorticoid pulse therapy. The most commonly used immunosuppressant was CsA (12 patients; 60%). Other prior treatments included IVIG (55%), rituximab (20%), interleukin-11 (15%) and TPO (60%). All patients underwent bone marrow biopsy, which showed megakaryocytosis in 12 (60%), megakaryocytopenia in 5 (25%) and normal megakaryocyte numbers with cell dysmaturity in 4 (20%). The characteristics of the study population are provided in Table 1 (1).

**Table 1.** Patient characteristics and treatment effectiveness of eltrombopag

Patient	Ageyares	Sex	Primary disease	Positive serological indexes	Duration	Duration of ITP	Max dose of steroids	Previous treatments	Lowest PLT count(×10^9^/L)	Bone marrow examination	Complications	Dose of eltrombopag	PLT counts at initiation (×10^9^/L)	Dose of steroids at initiation	Dose of immunosuppressants at initiation	Duration of eltrombopag (weeks)	Response	Time to response (days)	Treatments at the end of follow-up	Adverse events
1	58	F	SS	ANA1:160, SSA, Ro52, dsDNA, AnuA, AMA-M2,	16 years	2 weeks	Dex 30 mg/day	CsA, IVIG, TPO	5	Megakaryocytosis	Pancytopenia	25 mg/day	13	MP 40 mg/day	CsA150 mg/day	15	CR	5	Pred 7.5 mg/day	No
2	52	F	SLE	ANA1:320, SSA, Ro52	4 years	8 months	Mp 40 mg/day	RTX, LEF, HCQ	5	Megakaryocytopenia	No	25 mg/day	28	MP 40 mg/day	Tripterygium glycosides	2	NR	–	MP 36 mg/day	No
3	50	F	SS	ANA1:80, SSA, Ro52	3 years	3 years	Dex 40 mg/day	IVIG, TPO	4	Megakaryocytosis	No	25 mg/day	19	Pred 30 mg/day	–	4	CR	6	Pred 25 mg/day	No
4	63	F	SLE	ANA1:320, dsDNA, AnuA	2 months	2 months	MP 500 mg/day	CsA, IVIG, TPO, IL-11	2	ITP	Pancytopenia	25 mg/day	7	MP 40 mg/day	CsA150 mg/day	87	CR	20	Pred 5 mg/day, CsA50 mg/day	No
5	69	F	SS	–	5 years	2 years	MP 80 mg/day	CsA, HCQ, IVIG, TPO, IL-11, RTX	4	Megakaryocytosis	No	25/50 mg/day	11	Pred 30 mg/day	CsA200 mg/day	59	CR	7	MMF1g/day	No
6	66	F	SLE	ANA1:320, dsDNA, SSA, SSB	2 years	2 months	MP 40 mg/day	CsA, HCQ, RTX, IVIG	5	ITP	Membranous nephropathy	25 mg/day	21	Pred 30 mg/day	CsA100 mg/day, HCQ0.4g/day	104	CR	12	Pred5 mg/day, HCQ100 mg/day	No
7	62	F	UCTD	ANA1:3200, CENPB	10 years	14 months	MP 40 mg/day	IVIG	8	Megakaryocytosis	Cardiac insufficiency	50 mg/day	25	MP 40 mg/day	–	9	NR	–	MP28 mg/day	No
8	60	F	UCTD	ANA1:80, Ro52, AMA-M2	10 months	10 months	Dex 10 mg/day	CsA, IVIG, TPO	2	ITP	No	25 mg/day	2	Dex 10 mg/day	CsA200 mg/day	75	CR	49	CsA150 mg/day	No
9	71	F	SS	ANA1:640, SSA, Ro52, CENPB, dsDNA	2 years	2 years	MP 1000 mg/day	CsA, IVIG, TPO	0	Megakaryocytosis	Moderate anaemia	50 mg/day	2	–	CsA200 mg/day	13	PR	14	CsA250 mg/day	No
10	83	F	SS	ANA1:320, SSA, Ro52, CENPB	10 years	2.5 years	MP 40 mg/day	CsA, TPO	1	Megakaryocytopenia	Pancytopenia	25 mg/day	8	–	CsA300 mg/day	65	CR	56	CsA150 mg/day	No
11	72	F	SS	ANA1:320, SSA, Ro52, RNP	6 years	2 weeks	MP 40 mg/day	CTX, TPO	17	Megakaryocytopenia	Pancytopenia	25 mg/day	22	Pred 5 mg/day	–	2	NR	–	Pred5 mg/day	No
12	31	F	SS	ANA1:640, SSA, Ro52	3 years	3 years	Dex 40 mg/day	HCQ, IVIG, TPO, CsA, RTX	1	ITP	No	50 mg/day	8	MP 80 mg/day	HCQ0.4g/day, FK506 2 mg/day	12	CR	7	Pred 20 mg/day, HCQ0.4g/day, FK506 2 mg/day	No
13	58	F	SS	ANA1:80, SSA, Ro52	8 months	8 months	Pred 50 mg/day	HCQ, CsA	5	Megakaryocytopenia	No	25 mg/day	27	Pred 30 mg/day	HCQ0.2g/day	24	CR	14	Pred 5 mg/day, HCQ0.2g/day	No
14	41	F	UCTD	ANA1:320	1.5 years	1.5 years	Dex 40 mg/day	HCQ, CsA	7	Megakaryocytosis	No	50 mg/day	29	Pred 60 mg/day	CsA100 mg/day,	4	CR	14	Pred 40 mg/day	No
15	33	F	SLE	ANA1:1280, Sm, Ro52	9 months	9 months	Dex 40 mg/day	IVIG, TPO	1	Megakaryocytosis	No	25 mg/day	1	Pred 50 mg/day	–	12	CR	7	Pred 10 mg/day,	No
16	28	F	SS	ANA1:320, SSA, Ro52	12 years	12 years	MP 60 mg/day	CsA, HCQ,	5	Megakaryocytosis	No	50 mg/day	9	Pred 15 mg/day	CsA200 mg/day, HCQ0.4g/day	12	CR	28	CsA200 mg/day, HCQ0.4g/day	No
17	79	F	UCTD	ANA1:640	2 weeks	2 weeks	Dex 40 mg/day	TPO	2	Megakaryocytosis	No	25 mg/day	2	Pred 60 mg/day	–	2	CR	14	Pred 30 mg/day	No
18	21	F	SLE	ANA1:320, dsDNA	2 years	2 years	Dex 40 mg/day	IVIG, TPO	2	Megakaryocytosis	Hyperthyreosis	50 mg/day	2	MP 40 mg/day	HCQ0.4g/day	4	CR	10	Pred 20 mg/day	No
19	70	M	UCTD	ANA1:80, SSA, Ro52, SSB	4 months	4 months	Dex 10 mg/day	CsA, RTX, IL-11	4	Megakaryocytosis	No	75 mg/day	8	Dex 10 mg/day	CsA100 mg/day	4	NR	–	Pred 40 mg/day, CsA100 mg/day	No
20	30	F	UCTD	ANA1:80, Ro52	3 months	3 months	Dex 40 mg/day	IL-11	4	Megakaryocytosis	No	25 mg/day	4	Pred 60 mg/day	–	4	NR	–	Pred20 mg/day	No

F: female; M: male; SS: Sjögren’s syndrome; SLE: systemic lupus erythematosus; UCTD: undifferentiated connective tissue disease; ANA: antinuclear antibody; ANuA: anti-nucleosome antibody; CENPB: anti-centromere protein B antibody; AMA-M2: anti-mitochondrial M2 antibody; RNP: anti-ribonuclear protein antibody; Dex: dexamethasone; MP: methylprednisolone; CsA: ciclosporin A; IVIG: intravenous immunoglobulin; TPO: thrombopoietin; RTX: rituximab; LEF: leflunomide; HCQ: hydroxychloroquine; IL-11: Interleukin 11; CTX: Cyclophosphamide; ITP: idiopathic thrombocytopenia; PLT: platelet; Pred: prednisolone; HCQ: hydroxychloroquine; CR: complete response; NR: non-response; PR: partial response; MMF: mycophenolate mofetil.

### Risk factors for thrombocytopenia in CTD

There were no significant differences in gender, age, or course of disease between the 20 CTD patients with ITP and the 40 patients without ITP. Non-erosive arthritis (10% *vs* 55%, *P *=* *0.001), ILD (5% *vs* 38.24%, *P *=* *0.040) and haemoglobin values (97.25 ± 22.14 *vs* 116.90 ± 18.85, *P *=* *0.002) were higher in group CTD-ITP compared with CTD in univariate analysis (see Supplementary Table S1, available at *Rheumatology Advances in Practice* online). Logistic regression analysis was performed for all the above statistically significant factors. We found that that anaemia (OR = 8.832, *P *=* *0.007) was associated with increased risk of ITP, while non-erosive arthritis (OR = 0.045, *P *=* *0.001) and ILD (OR = 0.075, *P *=* *0.031) were associated with reduced risk (see Supplementary Table S2, available at *Rheumatology Advances in Practice* online).

### Effectiveness of eltrombopag

**Figure 1.**
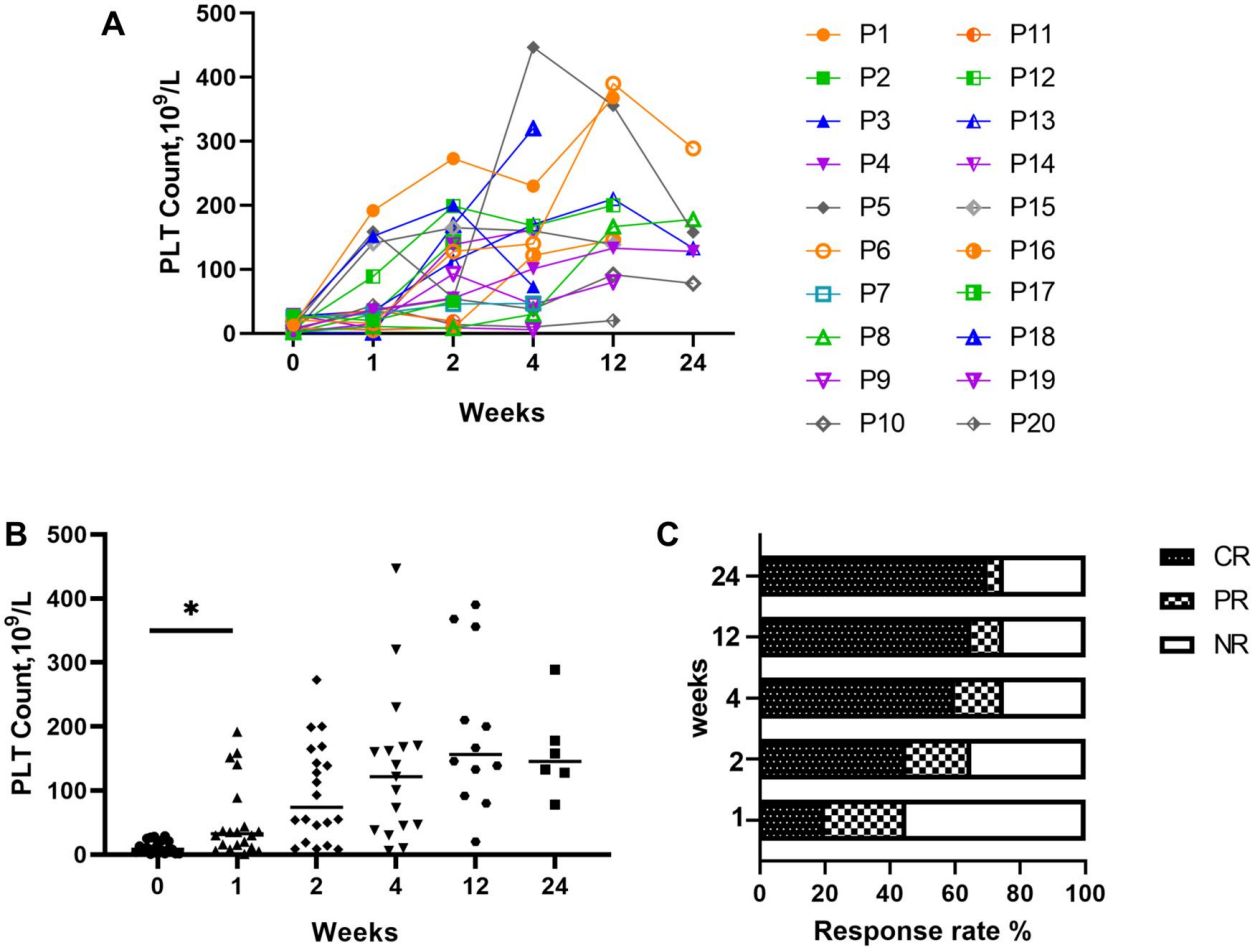
Efficacy of eltrombopag. (A) Platelet count trends in 20 patients after treatment with eltrombopag. (B) Changes in median platelet levels at different time points. Horizontal black lines in boxes indicate medians. (C) Each column represents the response rate at 1 week, 2 weeks, 4 weeks, 12 weeks and 24 weeks of treatment

In non-responders to glucocorticoids, immunosuppressants and/or other treatments, eltromopag was added to conventional therapy. The initial dose of eltrombopag was 25 mg/day in 13 patients, 50 mg/day in 6 and 75 mg/day in 1. The median platelet count at the commencement of treatment with eltrombopag was 8.5 (IQR, 2.5–21.75) × 10^9^/l. Eighteen patients received a median dose of prednisone equivalent to 50 (IQR, 30–60) mg, while 2 refused corticosteroid treatment as previous treatment had led to osteoporotic fragility fractures. The median platelet counts were 32.5 (IQR 12–77.75) × 10^9^/l at week 1, 74 (IQR 25.75–159.5) × 10^9^/l at week 2, 122 (IQR 41.5–169.0) × 10^9^/l at week 4, 156.5 (IQR 102.3–319.5) × 10^9^/l at week 12 and 145.5 (IQR 115.5 –205.8) × 10^9^/l at week 24 of eltrombopag treatment. The platelet count began to increase (*P *<* *0.05) after 1 week of eltrombopag treatment. Four patients (20%) achieved CR during the first week, and 9 (45%) in the second week. By 24 weeks of treatment, 14 patients (70%) had CR and 1 (5%) had PR resulting in an overall response rate of 75%. The median response time was 14 (7–20) days. All the patients who responded did so within 10 weeks (Supplementary Fig. S1, available at *Rheumatology Advances in Practice* online). At the end of the follow-up, the maintenance steroid dose was tapered down to 5 (IQR, 0–20) mg equivalented of prednisone. No adverse events and no thrombotic events related to eltrombopag were observed. Responses to treatment are shown in Table 1 (2), Fig. 1 and Supplementary Fig. S1, available at *Rheumatology Advances in Practice* online.

## Discussion

In clinical trials and observational studies, eltrombopag is demonstrated to be an effective and well-tolerated therapeutic for the treatment of primary ITP [9]. However, the effectiveness and safety of eltrombopag profiles in CTD-ITP patients are uncertain. As such, we evaluated this in a cohort of CTD-ITP patients. Our results show that eltrombopag has high effectiveness and a short onset to therapeutic effect. The study included 20 patients; of these, 14 had a satisfactory treatment effect, thus achieving an overall CR of 70%. This is generally consistent with previous reports on the effectiveness of eltrombopag in the treatment of primary ITP [9]. In our study, the shortest onset time of eltrombopag was 5 days, the median onset time was 14 days, and 9 cases (45%) took effect within 2 weeks. Median platelet counts remained stable after 1 month of eltrombopag treatment.

In our cohort, we analysed risk factors for CTD with thrombocytopenia. We found that CTD-ITP patients had fewer clinical manifestations of non-erosive arthritis and ILD, indicating that CTD patients with non-erosive arthritis and ILD would be at lower risk of thrombocytopenia events. However, more research is needed to elucidate the underlying mechanisms underlying this relationship. In addition, our data showed a higher proportion of anaemia in CTD patients with ITP, which correlated its occurrence. One study reported that iron might play a key role in thrombocytopenia [17]. In addition, bleeding due to thrombocytopenia can also promote anaemia [18]. As such, our identified risk factors for ITP in CTD are generally consistent with previous studies [17, 18].

Compared with primary ITP, the risk of infection in CTD patients is much higher, because of the immunosuppressive nature of existing therapies. Therefore, TPO-RA offers an attractive alternative as a non-immunomodulatory agent, which can be used to spare glucocorticoid and immunosuppressant administration, thereby reducing the risk of infection.

We identified no serious adverse reactions in this study. Studies have shown that patients with anti-phospholipid syndrome can form thrombi including pulmonary emboli after eltrombopag treatment [19]. A study by Guitton *et al.* suggested that if ACL is positive, alternative treatment should be considered if possible before TPO-RAs initiation in patients with SLE [20]. Therefore, our study excluded patients who were positive for ACL, especially with definite APS. To accurately profile the adverse risk profile in the CTD-ITP population, especially that of rare events, large-high-quality clinical studies are needed.

There are some limitations to our study. First, due to its retrospective design, some data were incomplete. Second, because all patients were recruited from a single center, there may be selection bias and the results may not be generalizable to all populations. Third, because of the limited sample size, our conclusions need to be validated by larger multicentre studies. Finally, the follow-up period was relatively short and we did not have sufficient data to evaluate whether ITP relapses after discontinuation of eltromopag.

In conclusion, we conducted a retrospective study of CTD-ITP patients to find that eltrombopag is effective and well-tolerated in refractory CTD-ITP. It has rapid onset, no immunosuppressive effects and a low risk of promoting infections. The long-term effectiveness and safety of eltrombopag in patients with CTD-lTP and its effect on CTD disease need to be verified in large multicentre clinical studies.

## Supplementary Material

rkae029_Supplementary_Data

## Data Availability

The data underlying this article will be shared on reasonable request to the corresponding author.
