# Supplementary material for: Effectiveness and safety of eltrombopag in connective tissue disease patients with refractory immune thrombocytopenia: a retrospective study
Source: Rheumatol Adv Pract. 2024 Mar 4;8(2):rkae029. doi: 10.1093/rap/rkae029 (PMC10942842; doi:10.1093/rap/rkae029)
Supplement: rkae029_Supplementary_Data [file rkae029_supplementary_data.doc]

**Supplementary Table S1** Comparison of clinic characteristics of CTD patients with ITP and non-ITP

|  | CTD-ITP(n=20) | CTD(n=40) | *p* value ^a^ |
| --- | --- | --- | --- |
| Female: Male | 19:1 | 38:2 |  |
| Age | 54.85±18.36 | 53.55±16.40 | 0.791 |
| Duration of protopathy (years) | 4.00±4.54 | 4.01±4.68 | 0.990 |
| **Comorbidity** |  |  |  |
| Hypertension | 6 | 10 | 0.918 |
| Diabetes mellitus | 3 | 2 | 0.409 |
| Thyroid diseases | 5 | 6 | 0.555 |
| Coronary artery disease | 2 | 4 | 0.648 |
| **Clinical manifestations** |  |  |  |
| Rash | 3 | 10 | 0.580 |
| Xerostomia | 5 | 21 | 0.080 |
| Xerophthalmia | 8 | 21 | 0.523 |
| Raynaud | 4 | 10 | 0.914 |
| Arthritis | 2 | 22 | 0.001 |
| Mucosal ulcer | 1 | 2 | 0.530 |
| Fever | 3 | 6 | 0.701 |
| **Organ involvement** |  |  |  |
| Serositis | 1 | 3 | 0.855 |
| Proteinuria | 2 | 3 | 0.869 |
| Central nervous system | 0 | 2 | 0.799 |
| Interstitial lung disease | 1 | 13 | 0.040 |
| Pulmonary arterial hypertension | 1 | 3 | 0.855 |
| **Laboratory examination** |  |  |  |
| WBC(×10^9^/L) | 6.39±3.13 | 6.11±2.61 | 0.733 |
| Hemoglobin (g/L) | 97.25±22.14 | 116.90±18.85 | 0.002 |
| Complement C3 (mg/dl) | 81.91±37.71 | 80.18±25.25 | 0.864 |
| Complement C4 (mg/dl) | 15.15±7.573 | 16.02 ±6.79 | 0.684 |
| Immunoglobulin G (mg/dl) | 1368±397.5 | 1624 ±594.7 | 0.057 |
| Immunoglobulin A (mg/dl) | 284.2±209.6 | 314.8±162.6 | 0.581 |
| Immunoglobulin M(mg/dl) | 145.9±137.9 | 174.1±290.5 | 0.618 |
| **Positive antibody** |  |  |  |
| Anti-SSA | 11 | 20 | 0.927 |
| Anti-SSB | 2 | 7 | 0.701 |
| Anti-dsDNA | 5 | 11 | 0.918 |
| Anti-RNP | 2 | 12 | 0.161 |
| Anti-CENPB | 3 | 5 | 0.893 |
| Anti-Ro52 | 13 | 22 | 0.643 |

^a^ *p* < 0.05 was considered as statistically significant.

Appendix:

Rash: Malar rash or cutaneous vasculitis (palpable purpura, microinfarcts, livedo reticularis)

Xerostomia: either (1) have a daily feeling of dry mouth for more than 3 months? OR (2) frequently drink liquids to aid in swallowing dry food

Xerophthalmia: ≥1 of (1) have daily, persistent, troublesome dry eyes for more than 3 months, (2) have a recurrent sensation of sand or gravel in the eyes, (3) use tear substitutes more than three times a day

Raynaud: Self-reported or reported by a physician, with at least a 2-phase colour change in finger(s) and often toe(s) consisting of pallor, cyanosis, and/or reactive hyperemia in response to cold exposure or emotion; usually one phase is pallor.

Arthritis: either (1) synovitis involving two or more joints characterised by swelling or effusion OR (2) tenderness in two or more joints and at least 30 min of morning stiffness

Oral ulcers: Oral ulcers observed by a clinician

Fever: Temperature >38.3°C

Serositis: Imaging evidence (such as ultrasound, X-ray, CT scan, MRI) of pleural or pericardial effusion, or both

Proteinuria: Proteinuria >0.5 g/24 hours by 24 hours urine or equivalent spot urine protein-to-creatinine ratio

Central nervous system: Delirium, psychosis or seizure

Interstitial lung disease: Pulmonary fibrosis seen on high-resolution CT or chest radiography, most pronounced in the basilar portions of the lungs, or occurrence of ‘Velcro’ crackles on auscultation, not due to another cause such as congestive heart failure.

Pulmonary arterial hypertension: Pulmonary arterial hypertension diagnosed by right-sided heart catheterisation according to standard definitions

**Supplementary Table S2** Logistic regression analysis of variables associated with CTD-ITP

|  | Regression coefficient | p-value | Wald value | OR value | 95% CI |
| --- | --- | --- | --- | --- | --- |
| Arthritis | -3.102 | 0.001 | 10.669 | 0.045 | 0.007~0.289 |
| ILD | -2.596 | 0.031 | 4.650 | 0.075 | 0.007~0.789 |
| Anemia | 2.178 | 0.007 | 7.404 | 8.832 | 1.839~42.410 |


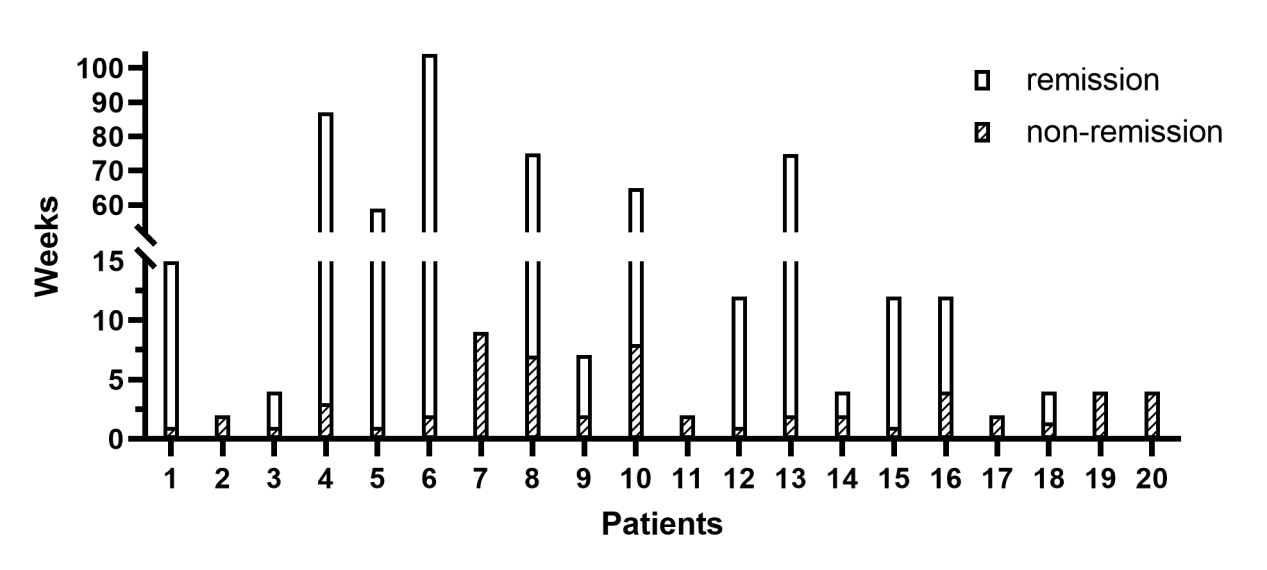


**Supplementary Figure S1** The response times of eltrombopag in total durations of therapy respectively.
